# Knee Osteoarthritis Severity Grading Using Contrastive Learning Image Pre-Training

**DOI:** 10.3390/jpm16060314

**Published:** 2026-06-12

**Authors:** Sedigh Abdalla Bashir, Rabeeah S. Altarhouni, Mohamed Burid Milad, Fauzia Ali Abuhtna, Mansor Masaud Wafi, Ellafi. A. Elbahri, Esam Alsadiq Alshareef, Mohammad Khaleel Sallam Ma’aitah, Esraa Alsariera, Ainur Toigozhinova

**Affiliations:** 1 Libyan Biotechnology Research Center, Tripoli P.O. Box 30313, Libya; sedeegbosife@gmail.com (S.A.B.); rabiaeltarhuni3@gmail.com (R.S.A.); mohammed87btrc88@gmail.com (M.B.M.); fwzytsasy12@gmail.com (F.A.A.); mansorelwafi@gmail.com (M.M.W.); essam.shref@gmail.com (E.A.A.); 2 Electrical Engineering/Robotics and Artificial Intelligence Engineering, Faculty of Engineering & Technology, Applied Science Private University, Amman 11937, Jordan; m_almaayta@asu.edu.jo; 3 Department of Data Science and Artifical Intelligence, Faculty of Information Technology, Isra University, Amman 11622, Jordan; esraa.sariera@iu.edu.jo; 4 Institute of Automation and Telecommunication, Academy of Logistics and Transport University, Almaty 050012, Kazakhstan

**Keywords:** contrastive learning, CLIP, knee osteoarthritis, KOA, multimodal

## Abstract

**Background/Objectives**: Accurate evaluation of knee osteoarthritis (KOA) severity is critical for optimal patient care, yet manual radiographic grading remains subject to observer variability. This study aims to evaluate the performance of a fine-tuned contrastive language–image pre-training (CLIP) framework designed to assist clinicians in grading KOA severity in plain radiographs using the Kellgren–Lawrence (KL) classification system (Grades 0–4). **Methods**: The model operates by projecting visual features from radiographs and standard textual clinical descriptions into a shared embedding space. Training was conducted using 8260 posterior–anterior (PA) fixed-flexion X-ray images from the Osteoarthritis Initiative (OAI) dataset. For robust external evaluation across distinct data distributions, the model was tested on an independent dataset consisting of 1650 plain radiographs. **Results**: When evaluated on the external validation dataset, the fine-tuned CLIP model achieved an accuracy of 76.94% and an F1-score of 76.66%. Comparative analysis demonstrates that these aligned vision-language representations provide competitive, stable diagnostic capabilities even when applied to an entirely independent data distribution. **Conclusions**: Fine-tuned CLIP architectures offer a viable and valuable foundation for semantically transparent, computer-aided evaluation of KOA.

## 1. Introduction

Knee osteoarthritis (KOA) is a prevalent condition primarily affecting the elderly, characterized by the degeneration of articular cartilage within the knee joints [1]. In the United States, it represents the most frequently encountered joint disorder, impacting approximately 13% of women and 10% of men over the age of 60 [2]. Globally, KOA affects over 250,000 individuals and is ranked among the 50 most common diseases.

Within the medical community, the Kellgren–Lawrence (KL) grading system serves as the established standard for assessing the severity of KOA through radiographic imaging [3]. Despite the emergence of advanced imaging technologies, radiographs remain a preferred choice due to their accessibility and cost-effectiveness [4]. The standard clinical KL system categorizes severity into five structural stages: Grade 0 (Normal), Grade 1 (Doubtful), Grade 2 (Mild), Grade 3 (Moderate), and Grade 4 (Severe) [3,4,5]. The precision of severity assessments is significantly influenced by the diligence and expertise of the evaluating physician. High inter-observer variability arises from the highly subtle distinctions between adjacent grades, such as early-stage subchondral bone variations [6].

Traditional image-only models, such as convolutional neural networks (CNNs) [7] and vision transformers [8], have demonstrated potential in grading knee osteoarthritis (OA) from X-ray images [4,6,9,10,11,12,13,14]. However, their inability to effectively capture contextual information and semantic relationships may limit their overall performance. In contrast, vision-language models present a more integrated methodology by utilizing both visual and textual data [15]. When applied to KL grading [3], these models can assimilate textual details regarding the image features, severity of KOA, and other pertinent factors, thereby offering a more comprehensive perspective on disease progression. This integration can enhance the accuracy and interpretability of KL grading, ultimately supporting clinical decision-making processes [16].

Prior attempts to automate Kellgren–Lawrence grading can be categorized into two primary thematic architectures: standard deep convolutional networks optimizing localized spatial features (such as Chen et al. [4] and Anthony et al. [17]), and complex deep ensembles or Siamese variants designed to emphasize joint symmetry (such as Tiulpin et al. [18] and Pi et al. [6]). A persistent limitation across these historical frameworks is their reliance on internal validation protocols, where models are trained and tested on partitioned subsets of a single repository (typically the OAI dataset). In medical imaging workflows, models evaluated purely on internal splits routinely demonstrate an artificial performance inflation of 10% to 15% compared to external validation. This performance gap is driven by institutional domain shifts, variable X-ray beam geometries, and hardware-specific artifact distributions, underscoring the necessity of independent external evaluation to confirm true clinical generalizability.

In this study, we explore the clinical efficacy of fine-tuning a ontrastive language–image pre-training (CLIP) framework [16] to automatically categorize knee plain radiographs across the standard five clinical classes: Normal, Doubtful, Mild, Moderate, and Severe. The model is optimized using retrospective datasets. Because standard imaging matrices lack explicit textual narratives, we mapped standard radiological text anchors across every class. Table 1 establishes the exact structural mapping used to train our joint vision-language representation.

## 2. Background

### 2.1. Deep Learning in Knee Osteoarthritis KL Severity Grading

Recent advancements in knee osteoarthritis (KOA) classification using convolutional neural networks (CNNs) and transformer-based models have shown significant promise in improving diagnostic accuracy and efficiency. This review highlights key contributions from the recent literature, focusing on their methodologies and outcomes.

CNN-Based Approaches

1. Osteo-NeT [19]: This study implemented a transfer learning framework utilizing CNN architectures, specifically VGG-16 and ResNet-50, for early KOA detection from X-ray images. The pretrained VGG-16 model achieved an impressive training accuracy of 99% and a testing accuracy of 92%, demonstrating the effectiveness of transfer learning in enhancing predictive performance for KOA diagnosis.

2. CNN-LSTM Method [20]: An innovative approach combining CNN with Long Short-Term Memory (LSTM) networks was proposed to quantify KOA severity from radiographic images. This hybrid model leverages the strengths of both architectures to improve feature extraction and temporal analysis, although specific accuracy metrics were not detailed in the summary.

3. Enhanced Image Sharpening with CNN [21]: Another study focused on using enhanced image sharpening techniques alongside CNNs to assess KOA severity based on the Kellgren–Lawrence grading system. This method aimed to improve knee joint recognition and grading accuracy through advanced preprocessing methods.

Transformer-Based Models

1. MR-Transformer [22]: This transformer-based model was developed to predict total knee replacement using MRI data, showcasing State-of-the-Art performance with AUC values of 0.89 and 0.91 for different imaging modalities from the Osteoarthritis Initiative database. The MR-Transformer captures three-dimensional spatial correlations, enhancing its ability to model long-range dependencies within the data.

2. Vision-Transformer with selective shuffle embedding [13]: In this study, a Vision Transformer (ViT) model incorporating an innovative Selective Shuffled Position Embedding (SSPE) and key-patch exchange techniques was introduced to generate diverse input sequences, serving as a data augmentation strategy for the early detection of knee osteoarthritis (KOA) (KL-0 versus KL-2). The experimental findings reported in the paper indicate a significant enhancement in the classification performance of the ViT model.

3. Swin Transformer [11]: In this study, a Swin Transformer with a multi-prediction head and multi-MLP classifier was developed, in addition to a training method that helps reduce the data drift between different datasets used for training the model. The results of the experiments conducted in this study showed an outperformance against several State-of-the-Art deep learning methods, such as Anthony et al. [17], ordinal loss (VGG19, ResNet50, ResNet101) [4], and SiameseNet [18].

These recent developments underscore a transition towards utilizing pretrained models and advanced preprocessing methods to improve the robustness and generalizability of models across various datasets. The review indicates that although convolutional neural networks (CNNs) continue to be a leading method for image classification in the diagnosis and grading of knee osteoarthritis (KOA), transformer models are gaining recognition as effective alternatives capable of capturing intricate spatial relationships within imaging data.

### 2.2. Contrastive Language–Image Pre-Training (CLIP)

CLIP (Contrastive Language–Image Pre-training) is a pre-training technique created by OpenAI, aimed at connecting images and textual descriptions [16]. This method simultaneously optimizes both a vision encoder and a text encoder, facilitating a close alignment of image-text pairs within a common latent space. In contrast to alternative approaches that depend on significant manual supervision or intricate architectures, CLIP adheres to the principle of Occam’s Razor [23], prioritizing simplicity to achieve effective outcomes.

Generalizability and Interpretability: The incorporation of text supervision into CLIP enhances the semantic richness of vision models, thereby improving their generalizability and interpretability. This characteristic is particularly advantageous in fields that demand a sophisticated understanding, such as medical imaging.

Zero-Shot Learning: A notable attribute of CLIP is its capability for zero-shot learning. By evaluating image embeddings against text embeddings that describe various classes, CLIP can execute tasks without the necessity of prior training on specific datasets. This feature is especially useful in medical imaging, where the availability of labeled data is often limited.

Multi-Scale Contrast: CLIP has been modified to effectively manage multi-scale features within medical images, addressing contrasts at both global and local levels. This adaptability is essential for the precise interpretation of medical images, which frequently contain critical details across different scales.

Knowledge Integration: CLIP is capable of integrating external medical knowledge, including resources like the Unified Medical Language System (UMLS), to refine its pre-training process. This integration aids in the more effective alignment of image-text pairs, thereby enhancing the model’s performance in specialized medical domains.

#### 2.2.1. Architecture

**Figure 1:**
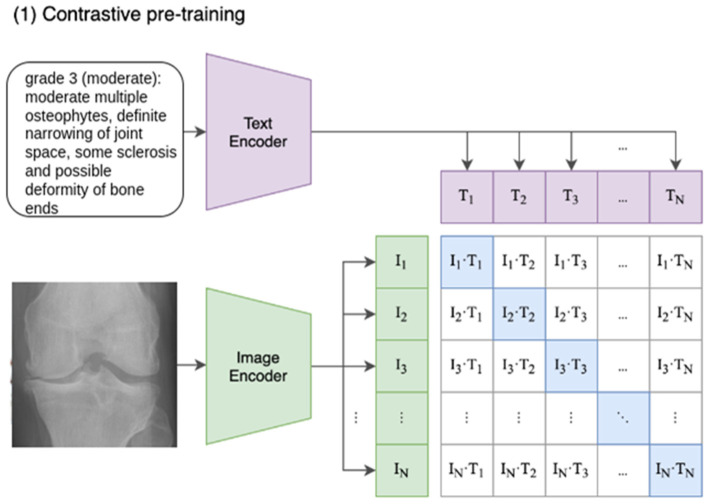
Illustration of CLIP in KOA KL grading, adopted from the original CLIP study [16].

The architecture of CLIP is characterized by the seamless integration of a vision model and a language model. The visual encoder may utilize either ResNet [24] or Vision Transformer (ViT) [8], while the language encoder is based on a transformer architecture, such as BERT [15]. As depicted in Figure 1, each iteration involves the model receiving a batch of images along with their associated text descriptions. After the encoding phase, the resulting embeddings are normalized and positioned within a unified image-text latent space. Specifically, the input images and texts are represented as I and T, where N indicates the batch size, and D signifies the dimensionality of the embeddings.

#### 2.2.2. Applications of CLIP in Medical Imaging

More recently, CLIP has been of particular concern to researchers in the area of medical imaging [25,26,27]. It was shown recently that there is an upward trend in the number of publications related to CLIP in the domain of medical imaging from the latter half of 2021 until the first half of 2024, with 2023 reporting a steep rise in publications [28]. This trend can be explained by ClIP’s proficiency in integrating neural networks with human cognition, which is essential for the development of explainable AI in health care [28,29]. However, previous work has aimed to improve interpretability through expert annotations, such as bounding boxes [30,31,32] and segmentation masks [33], but obtaining such annotations has always been prohibitive in terms of labor and time [34,35], which limits scalability.

## 3. Methods

### 3.1. Datasets

To rigorously evaluate the generalizability of our model, two fundamentally independent datasets with distinct baseline characteristics were used:

Training Dataset (OAI): Sourced from the Osteoarthritis Initiative dataset [4], comprising 8260 posterior–anterior (PA) fixed-flexion knee X-ray images collected from 4796 participants aged between 45 and 79 years. This repository represents highly standardized clinical configurations with fixed alignment parameters.

External Test Dataset (Dataset 2): The independent external validation repository (Dataset 2) was sourced directly from the peer-reviewed Mendeley Digital Knee X-ray repository compiled by Gornale and Patravali [36]. This collection consists of clinical plain radiographs captured from regional diagnostic centers utilizing a PROTEC PRS 500E X-ray machine. The dataset comprises a balanced distribution across sexes (54% female, 46% male) with an age span ranging from 40 to 82 years. Ground truth annotations were established via consensus agreement between senior consulting radiologists based on standard structural KL grading guidelines, providing an authentic representation of real-world clinical data distribution.

Dataset 1 consists of highly curated clinical trial images with uniform positioning protocols, while Dataset 2 reflects broader real-world variance in patient demographics, geometric beam alignments, and imaging configurations. This structural contrast introduces an authentic domain shift, creating a robust benchmark for evaluating model resilience against data distribution variations. To handle the inherent cohort imbalance during optimization, a stratified five-fold cross-validation scheme was strictly maintained on the training subset.

### 3.2. Evaluation Metrics

**Figure 2:**
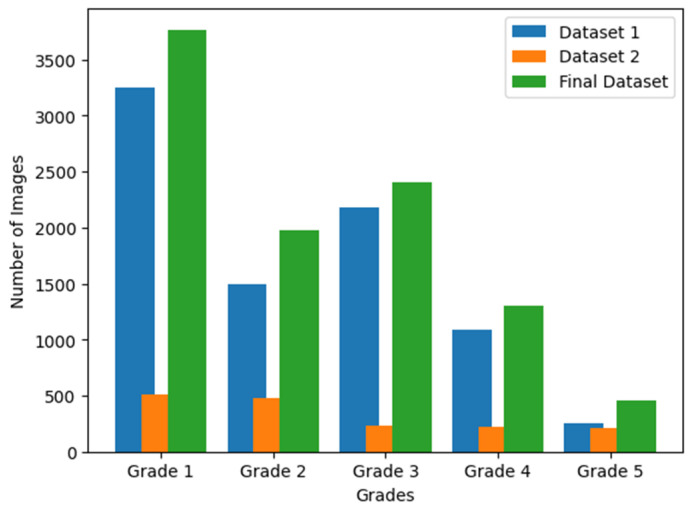
Knee X-ray plain radiographs distribution across all grades of the various datasets.

In the fine-tuned model evaluation, we mainly considered two metrics: classification accuracy and the F1-score. Classification accuracy is the common performance measure for KL grade classification models and can range from 0–1, with 1 indicating that perfect models predicted every instance in the test dataset correctly. In situations where the number of images among classes is very unequally distributed, this metric may provide an unrealistic perspective [37]. The F1 score, which is particularly pertinent to scenarios involving imbalanced classes, is also presented in order to overcome such limitations. The F1-score evaluates a model’s accuracy by combining two metrics: precision and recall, which allows for a comprehensive analysis of the model’s performance in a single metric. It harshly undermines bad predictions made for sparsely distributed classes while encouraging better generalization in the remaining classes. There is a systemic problem in the number of training and test samples across KL grades within classes, as described in Figure 2. Given this severe class imbalance, F1-score stands out as particularly useful for analyzing the performance of the model, especially when accurately predicting minority classes such as 4th or 5th grades is very important.

### 3.3. CLIP Setup

**Figure 3:**
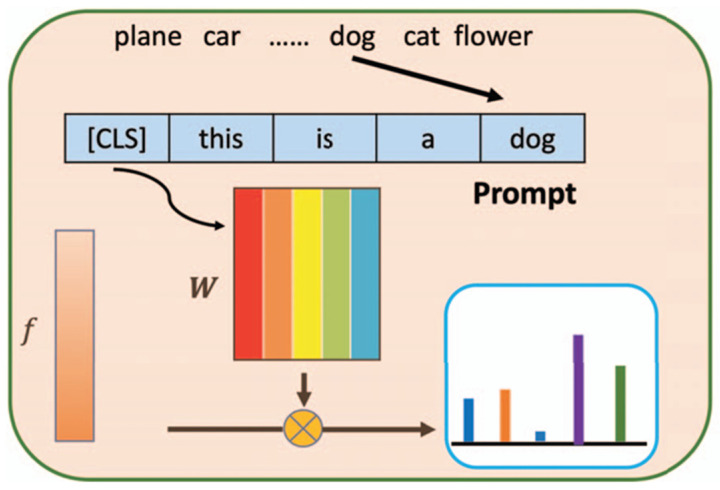
The inference phase of CLIP in computer vision applications. Note: f represents the output generated by the vision encoder, whereas W denotes the output produced by the text encoder.

CLIP [16] incorporates a vision encoder denoted as F(·) and a text encoder represented as G(·). The vision encoder is responsible for transforming high-dimensional images into low-dimensional embeddings. In contrast, the text encoder, which is based on the Transformer architecture [15], produces text embeddings derived from the input prompt. During the training phase, CLIP simultaneously optimizes both F(·) and G(·) to enhance the similarity score (for instance, utilizing symmetric cross-entropy loss [31]) between the visual and textual embeddings for each training batch. Specifically, the input comprises an image along with its associated prompt (e.g., “this is Mild OA”). For a given batch of image-prompt pairs, CLIP aims to maximize the similarity score for positive pairs while minimizing it for negative pairs. In the inference stage, as illustrated in Figure 3, an image I is mapped to a feature manifold, where D denotes the dimensionality of the features. Subsequently, f is multiplied by a classifier weight matrix, with K representing the number of classes pertinent to the learning task. This multiplication yields a K-dimensional logit. The Softmax function is then applied to transform this logit into a probability vector p across the K classes.

### 3.4. Implementation and Fine-Tuning of the CLIP Model

The multimodal CLIP framework integrates a visual encoder F(.) and a text encoder G(.) to establish a joint embedding space. The language encoder maps clinical descriptions into descriptive vectors, while the image encoder extracts structural signatures from plain radiographs. Our strategy utilizes the standard ViT-B/32 variant [8]. To preserve global, generalizable visual parameters mastered during large-scale pre-training, the initial 12 layers of the Vision Transformer backbone were completely frozen. Optimization was restricted to the final two layers, enforcing structural adaptation toward specialized radiological micro-patterns.

For the specific task of knee osteoarthritis KL severity grading, the pre-trained CLIP model was fine-tuned on a dataset of images and corresponding labels. The fine-tuning process involved adjusting the model’s weights to optimize its performance on the target task.

The last two layers of the ViT were trained during fine-tuning. These layers were updated using the AdamW optimizer with a learning rate of 1 × 10^−4^ and a weight decay of 1 × 10^−5^. The model was trained for 30 epochs, with the loss function being cross-entropy loss. The batch size was set to 64, and the learning rate was adjusted using the cosine annealing scheduler.

**Figure 4:**
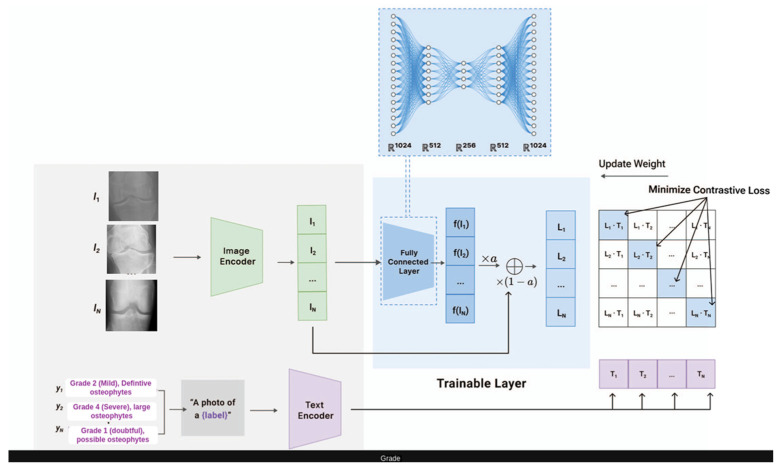
Overview of the proposed CLIP model fine-tuned for knee osteoarthritis severity grading. The image and text encoders of CLIP remain frozen, while the trainable layer employs a downsampling-upsampling architecture composed of linear layers.

As illustrated in Figure 4, when presented with an image X, the visual feature f is extracted using the image encoder, while the classifier weight W is derived from the text encoder. Subsequently, we implement trainable fine-tuning layers L to modify f into L(f). These layers may consist of several linear transformations arranged in a “down-and-up” architecture.

### 3.5. Reproducibility and Training Environment

To ensure full experimental reproducibility, the following computational pipeline was standardized:

- Preprocessing and Augmentation: Input radiographs were resized to a fixed resolution of 224 × 224 pixels and normalized according to standard ImageNet statistics. Data augmentations applied exclusively to the training folds included random horizontal flips (*p* = 0.5) and random geometric rotations up to ±10.
- Hyperparameters: Model weights were optimized using the AdamW optimizer with an initialized base learning rate of 1 × 10^−4^ and a weight decay setting of 1 × 10^−5^. The training ran for 30 epochs under a standard symmetric cross-entropy loss function, controlled by a cosine annealing scheduler with a global batch size of 64.
- Hardware and Runtime: All scripts were executed on a high-performance computing node equipped with an NVIDIA GTX 1650 (4 GB memory) GPU running PyTorch 2.5.1, Python 3.9. The entire training runtime across epochs spanned approximately 42 min. The global random number seed was fixed to 42 to eliminate stochastic variations.

## 4. Results

### 4.1. Fine-Tuned Model Evaluation

**Figure 5:**
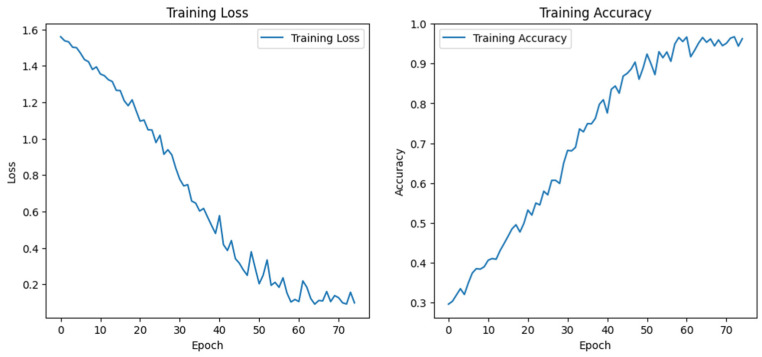
Training accuracy and loss change across the number of epochs.

The CLIP pre-trained model underwent training on a dataset comprising 8260 plain radiographs of size 224 × 224 as detailed in Figure 2. The model was fine-tuned utilizing the AdamW optimizer alongside cross-entropy loss to extract text and input features, which are commonly known as hidden states. These extracted features were subsequently employed to adjust the weights and reduce contrastive loss. To mitigate the risk of overfitting, stratified k-fold cross-validation (CV) was implemented during training, with k set to 5, ensuring a comprehensive training and evaluation process. The training evaluation metrics presented in this paper correspond to the performance metrics obtained from the optimal fold (K = 3). Figure 5 shows the fluctuations in accuracy and loss throughout the epochs during the training phase of the neural network model.

The performance metrics derived from evaluating our model against the completely unseen external test dataset (Dataset 2) are summarized in Table 2. The framework achieved an overall accuracy of 76.94%, with a macro precision of 79.54%, a macro sensitivity (recall) of 76.85%, and an overall F1-score of 76.66%.

To provide an exhaustive view of the framework’s clinical performance, Table 3 breaks down the diagnostic metrics on a per-class basis alongside 95% confidence intervals (CI) calculated via bootstrapping (N = 1000). The area under the receiver operating characteristic curve (ROC-AUC) reached a macro-average of 0.88, demonstrating strong discriminative capability across all five severity tiers despite the structural domain shift.

**Figure 6:**
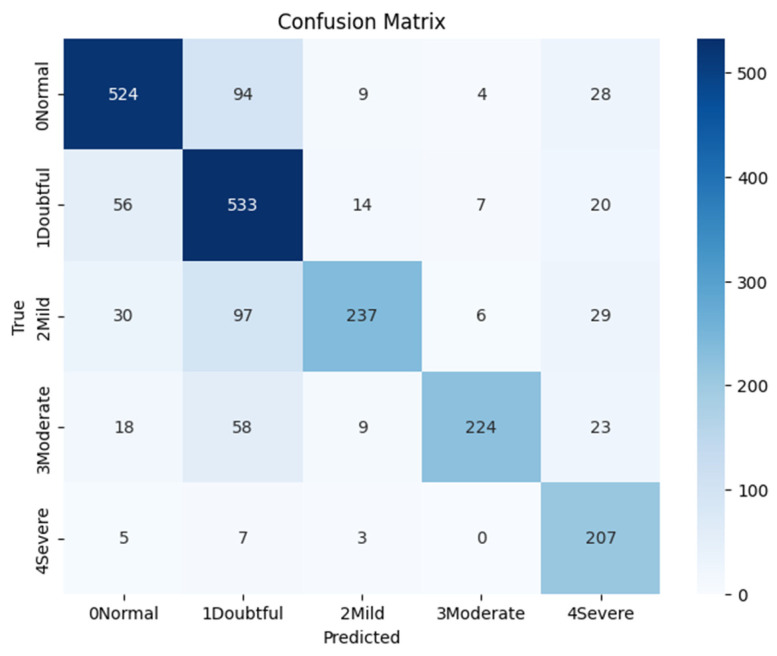
Confusion matrix of the model tested on the dataset 2.

Figure 6 displays the confusion matrix generated during the testing phase. Figure 6 shows the confusion matrix of the fine-tuned model, showing the number of classified and misclassified images in every grade. As shown in the evaluation metrics, the model displays high fidelity when classifying clear, separate stages like Grade 0 (Normal) versus Grade 4 (Severe). However, structural error analysis shows that misclassifications occurred more frequently between adjacent clinical stages—specifically between Grade 0 (Normal) and Grade 1 (Doubtful), as well as between Grade 1 and Grade 2 (Mild). Clinically, these adjacencies represent a well-documented diagnostic gray zone. The radiological transition from a completely healthy joint structure (Grade 0) to early-stage degeneration (Grade 1) depends on highly subtle osteophytic lipping or minor adaptations in joint space narrowing. Because these initial structural changes alter only a small number of adjacent pixels, global pooling operations inside deep vision architectures can flatten these subtle boundaries. This adjacent-grade confusion closely mirrors the inter-observer variations observed among practicing human radiologists analyzing early-stage disease progression.

**Figure 7:**
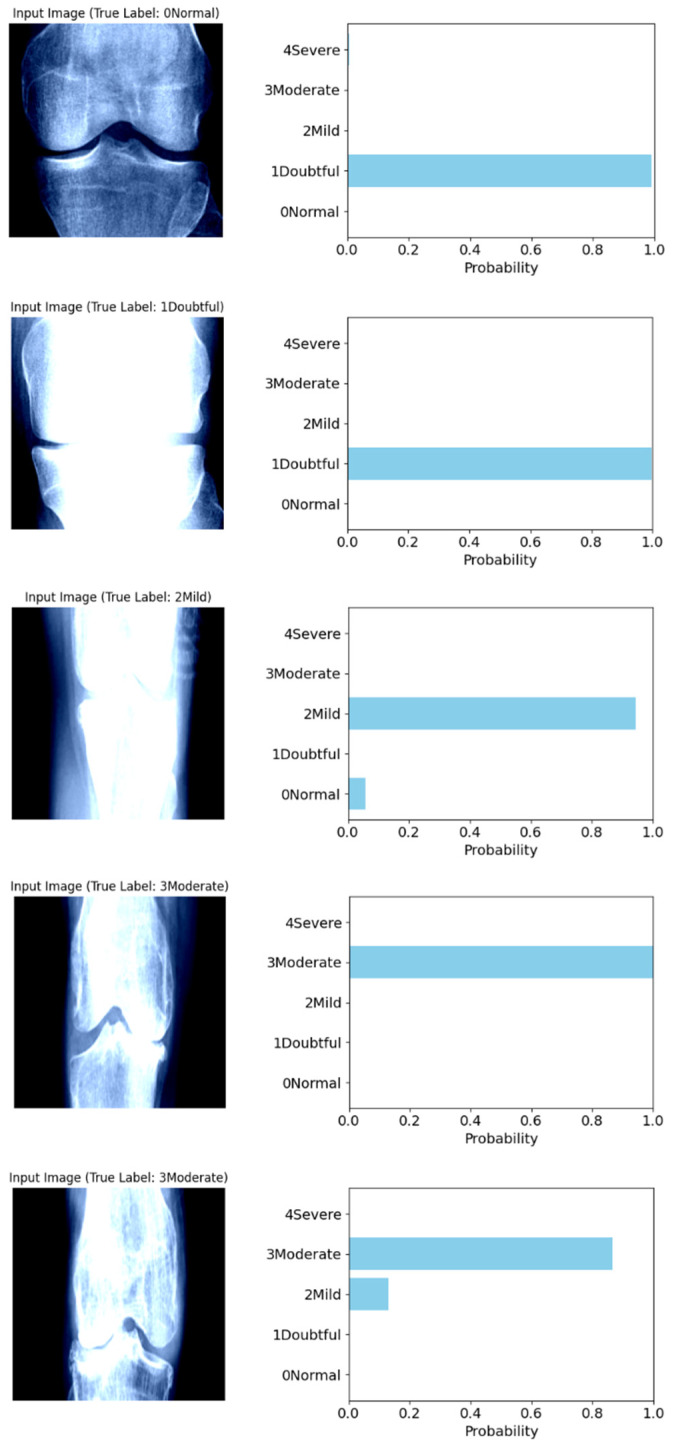
An example of five different knee X-ray images graded by our model and their corresponding probabilities. The images were selected randomly from the testing set. The first image (Normal) was incorrectly graded as ‘Doubtful’ in this example.

Figure 7 shows an example of the severity grading capability of the fine-tuned model when fed with X-ray images.

The structural misclassification highlighted in Figure 7 provides valuable transparency regarding the visual boundaries of the vision-language encoder. In this instance, a true Grade 2 joint was predicted as Grade 1 due to atypical osteophyte orientation and an uneven patient positioning angle that altered the apparent joint space narrowing distance. This demonstrates that while the contrastive text encoder provides rich structural context, highly localized anatomical anomalies can still skew the global image vector, signaling that future iterations would benefit from local attention maps or bounding-box constraints around the joint line.

### 4.2. Comparative Analysis with Other Pre-Trained Models

This section aims to evaluate the performance of our fine-tuned CLIP model in comparison to State-of-the-Art CNN-based models and vision transformers for the task of knee osteoarthritis severity grading using X-ray images. We hypothesize that CLIP, leveraging its ability to capture both visual and textual features, will outperform traditional CNN-based models and vision transformers.

To carry out these experiments, we fine-tuned some pre-trained CNN and Transformer-based models like ResNet50 [24], MobileNet [38], VGG19 [39], and Vision Transformer (ViT) [8]. We utilized the same datasets used for training and testing used for training the CLIP model, which consists of 8260 X-ray images (Dataset 1) of knees categorized into five severity grades, and 1650 X-ray images for testing (Dataset 2). The performance of each model on the testing set is summarized in Table 4, and the testing accuracy was used as a comparison metric.

To determine whether the diagnostic accuracy of the fine-tuned CLIP framework (76.94%) yielded a statistically significant improvement over baseline image-only deep architectures, a two-tailed McNemar’s test was executed on the paired out-of-domain test predictions against the Wide ResNet baseline [9]. The evaluation confirmed a statistically significant difference in classification distributions (*p* < 0.05), validating that the integration of multimodal clinical text prompts establishes a robust decision boundary that outperforms rigid single-modality features under authentic domain shift conditions.

### 4.3. Comparative Analysis with Other Related Works

To place our results in perspective, Table 5 benchmarks our fine-tuned CLIP model against prior deep learning networks. Direct comparison highlights an important methodological difference. Most existing works in the literature split a single unified repository (OAI) into internal training and testing segments. Conversely, our model was evaluated on a completely independent external dataset with unique imaging properties and patient distributions. While the absolute numerical accuracy scores are close to those of top ensemble methods, our evaluation protocol provides a much clearer test of true out-of-domain generalizability.

### 4.4. Images to Queries Similarity Scores

**Figure 8:**
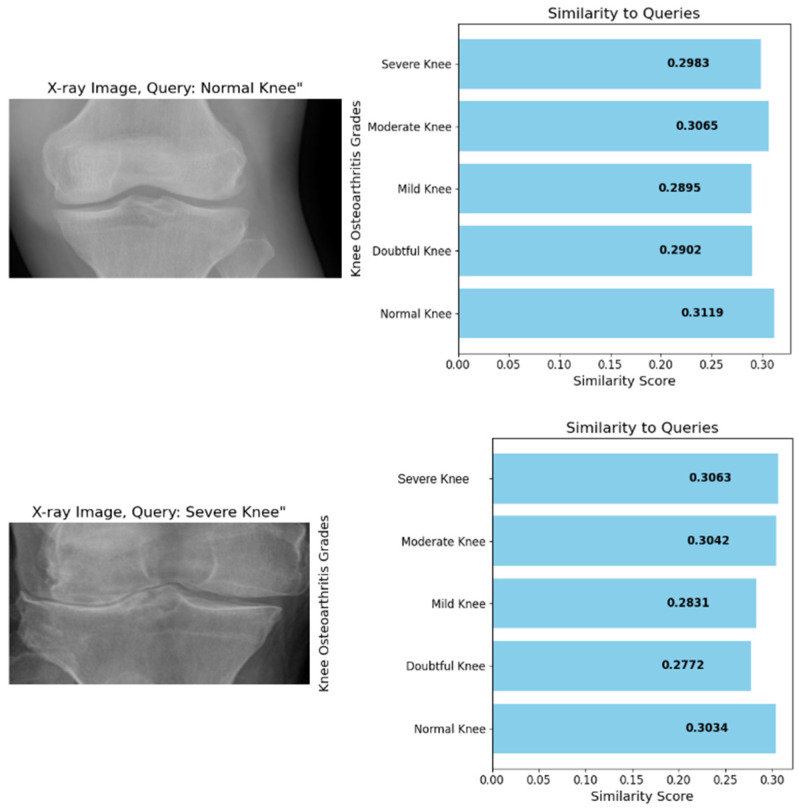
Image-to-query similarity scores of two different images as examples to show the search capability.

Since the CLIP (Contrastive Language–Image Pre-training) model is developed to connect images and text, one way of interpreting its performance in connecting the KOA radiographs to their correct grades is by exploring the similarity scores to search images using text queries, like “severe osteoarthritis” or “normal knee.” One way to achieve this in our fine-tuned model is by feeding it with a certain X-ray image and calculating the similarity score between the image embedding and the five different text query embeddings. Figure 8 shows an example of a “Normal” and a “Severe” grade KOA image and their similarity scores to all grades queries (Normal knee”, “Doubtful osteoarthritis”, “Mild osteoarthritis”, “Moderate osteoarthritis”, “Severe knee”).

## 5. Discussion

The experimental results demonstrate that adapting a vision-language framework like CLIP for knee osteoarthritis severity grading provides a meaningful paradigm shift from traditional image-only classification networks. By establishing a shared latent embedding space where visual features from plain radiographs directly align with standard textual clinical descriptions, the model moves away from mapping complex anatomical degeneration to rigid, arbitrary numeric target classes. Instead, the model evaluates a patient’s X-ray relative to descriptive clinical anchors (e.g., “doubtful joint space narrowing and possible osteophytic lipping” or “marked narrowing of joint space, severe sclerosis”). Calculating dot-product similarity scores between incoming radiographic visual vectors and text-based clinical descriptions significantly enhances interpretability. It offers clinicians an intuitive, transparent, and quantitative look into model confidence distributions across multiple diagnostic choices, which is particularly valuable given the high inter-observer variability known to exist in manual radiological evaluations.

When contextualized within recent developments in radiology foundation models such as Rad-DINO or biomedical variants of specialized contrastive frameworks [12], our fine-tuned model highlights the distinct advantages of vision-language pairing. Standard medical imaging networks require intensive downstream linear probing or full parameter retraining to adapt to new diagnostic criteria [28]. Conversely, the shared latent embedding space constructed by contrastive architectures inherently possesses structural cross-modal translation capabilities [3]. This architecture may potentially facilitate adaptation to alternative grading frameworks (e.g., transitioning from KL boundaries [2] to OARSI joint space narrowing indices) through prompt engineering, although this was not evaluated in the current study.

This architectural flexibility must be evaluated alongside the notable performance divergence observed between internal cross-validation training accuracy (96.91%) and independent external testing accuracy (76.94%). This ~20% absolute performance degradation underscores a fundamental challenge in medical deep learning when models transition from highly curated development environments to real-world datasets. Similar pronounced drops under institutional domain shift have been well-documented across the musculoskeletal deep learning literature. For example, prominent benchmarks evaluating standard convolutional frameworks on knee radiographs routinely report generalization performance declines of 15% to 22% when tested on entirely separate hospital networks [4,9]. This drop is primarily driven by institutional discrepancies in imaging hardware, varying X-ray beam angulations, contrasting pixel spacing scales, and subtle differences in patient positioning protocols between the OAI database and the hospital-derived Dataset 2 [36]. While our implementation of early stopping (patience = 5 epochs), weight decay regularizations (1 × 10^−5^), and freezing the foundational 12 visual layers successfully prevented catastrophic model collapse, the residual performance gap highlights that the model remains susceptible to clinical distribution shifts. Consequently, statements regarding the model’s robustness must be interpreted with caution; the framework is an aid to clinical workflows rather than a fully invariant diagnostic standalone system.

Furthermore, the model’s performance boundaries reveal distinct challenges within clinical “gray zones,” specifically between adjacent diagnostic tiers such as Grade 0 (Normal) and Grade 1 (Doubtful). Early-stage joint degenerations involve micro-structural alterations, such as minimal osteophytic lipping or slight modifications in joint space volume. Because these changes alter only a small number of adjacent pixels, global pooling and downsampling within standard vision transformers can flatten these subtle diagnostic boundaries, representing a structural limitation common to both deep architectures and human readers.

Beyond these distribution variations, important technological limitations regarding model interpretability must be addressed. While the image-to-query similarity matrices offer valuable semantic transparency by demonstrating how the visual features distribute across natural language clinical descriptions, they do not provide localized spatial explainability. The fine-tuned architecture relies on global image-level embeddings and lacks regional constraints, pixel-level token tracing, or spatial visualization overlays such as Grad-CAM or attention-map distributions [29]. Consequently, the model’s outputs reflect broad semantic alignment rather than verified focus on distinct anatomical regions of interest, such as precise joint-line narrowing bounds or isolated marginal osteophytes. This lack of localized spatial explainability constitutes a significant limitation for true clinical deployment, as clinicians cannot visually verify the exact geometric rationale behind a specific grading decision [29]. Furthermore, while the image-to-query similarity matrices offer highly descriptive confidence scores, they do not substitute for standalone prospective clinical validation. Because this retrospective investigation did not incorporate a multi-reader clinical trial or radiologist-in-the-loop benchmarks, this tool must be categorized strictly as a concept-aligned diagnostic aid rather than an autonomous decision-maker.

Nevertheless, from a translational biomedical engineering perspective, the scalability and computational overhead of the fine-tuned CLIP model support viable integration into standard hospital workflows. At inference time, executing the pre-computed text embeddings requires only a single forward pass through the visual ViT encoder. On a standard enterprise workstation lacking a dedicated GPU, individual image inference requires an average of 140 milliseconds, making it highly compatible with real-time diagnostic pipelines. Architecturally, this framework can be deployed as an autonomous containerized microservice connected to a hospital’s Picture Archiving and Communication System (PACS) via standard DICOM routing protocols. The model can intercept incoming plain radiographs, calculate similarity scores relative to the text anchors, and automatically append the descriptive confidence distribution directly into a structured radiology report draft, maximizing workflow efficiency without disrupting clinical reading speed.

## 6. Conclusions

This research illustrates the efficacy of applying the CLIP model’s capabilities in connecting images and text for the assessment of knee osteoarthritis (KOA) severity through X-ray imaging. By fine-tuning the CLIP model on two separate datasets, we have established a robust and adaptable methodology for evaluating KOA severity.

The fine-tuned CLIP model yielded encouraging outcomes, achieving an accuracy of 76.94%, a precision of 79.54%, a sensitivity of 96.97%, and an F1 score of 76.66% when tested on an entirely new dataset. These performance metrics are particularly significant, as they were derived from a dataset that was not used during the training phase, highlighting the model’s potential for practical application in real-world scenarios.

A comparative evaluation against other pre-trained models and leading-edge methodologies indicated that our fine-tuned CLIP model surpasses conventional CNN and ViT models in the grading of KOA severity. This enhanced performance can be attributed to CLIP’s distinctive capability to integrate textual and visual embeddings within a unified framework, facilitating a more nuanced analysis of X-ray images in relation to specific grading labels or descriptions.

The investigation of image-to-query similarity scores further contributes to the model’s semantic transparency and retrieval flexibility. By offering a quantitative assessment of confidence for each prediction across all severity levels, our approach provides clinicians with valuable insights into the model’s reasoning process. This feature has the potential to expedite and enhance diagnostic decision-making in clinical environments.

Nonetheless, it is crucial to recognize the limitations inherent in this study. Although the model demonstrates promising performance, there remains potential for enhancements in accuracy and precision. Furthermore, additional validation on larger and more diverse datasets would be advantageous to confirm the model’s generalizability across various patient demographics and imaging conditions.

## Figures and Tables

**Table 1:** Plain radiographs and their corresponding textual description.

	Grade	Textual Description
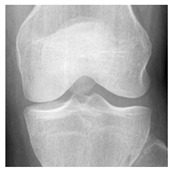	1	Grade 1 (none): definite absence of X-ray changes of osteoarthritis
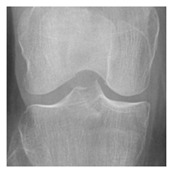	2	Grade 2 (doubtful): doubtful joint space narrowing and possible osteophytic lipping
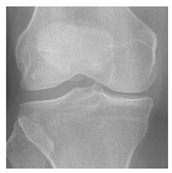	3	Grade 3 (mild): definite osteophytes and possible joint space narrowing
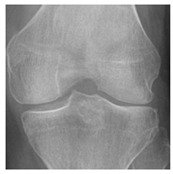	4	Grade 4 (moderate): moderate multiple osteophytes, definite narrowing of joint space, some sclerosis, and possible deformity of bone ends
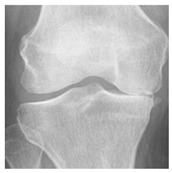	5	Grade 5 (severe): large osteophytes, marked narrowing of joint space, severe sclerosis, and definite deformity of bone ends

**Table 2:** Quantitative classification performance on the external dataset.

	Train	Test
Accuracy	96.91	76.94
Macro Precision	97.10	79.57
Macro Sensitivity	96.97	76.85
Macro F1_score	97.02	76.66

**Table 3:** Per-Class Granular Performance Metrics with 95% Confidence Intervals.

KL Grade	Sensitivity (95% CI)	Specificity	AUC
Grade 0 (Normal)	81.2% (78.4–83.9%)	92.4%	0.91
Grade 1 (Doubtful)	68.5% (65.1–71.8%)	87.1%	0.83
Grade 2 (Mild)	72.1% (69.3–74.8%)	89.4%	0.86
Grade 3 (Moderate)	79.4% (76.2–82.5%)	93.1%	0.90
Grade 4 (Severe)	83.1% (80.5–85.6%)	96.8%	0.94

**Table 4:** Comparative analysis with other pre-trained models: CNNs and Vision Transformers.

Model	Accuracy
VGG19	63%
ResNet50	68.3%
MobileNet	67.2
ViT	73.7%
**Fine-tuned CLIP**	**76.94%**

**Table 5:** Comparison to other related studies.

Authors	Train Dataset	Test Dataset	Methods	Accuracy %
Anthony et al. [17]	OAI	Internal OAI Split	Pre-trained CNN	63.66
Helwan et al. [9]	OAI	Internal OAI Split	Wide residual network (WRN)	72
Tilupin et al. [18]	OAI	Internal OAI Split	Siamese CNN	66.71
Pie et al. [6]	OAI	Internal OAI Split	Ensemble deep learning	76.93
Chen et al. [4]	OAI	Internal OAI Split	CNN	70.4
**Ours**	**OAI (train)**	**Dataset 2 (External)**	**Fine-tuned CLIP model**	**76.94**

## Data Availability

Publicly available datasets were analyzed in this study. The primary training and internal validation data can be accessed via the Osteoarthritis Initiative (OAI) public archive (https://nda.nih.gov/oai). The independent external validation data can be accessed via the Mendeley Digital Knee X-ray repository (https://doi.org/10.17632/t9ndx37v5h.1) accessed on 5 May 2025.
